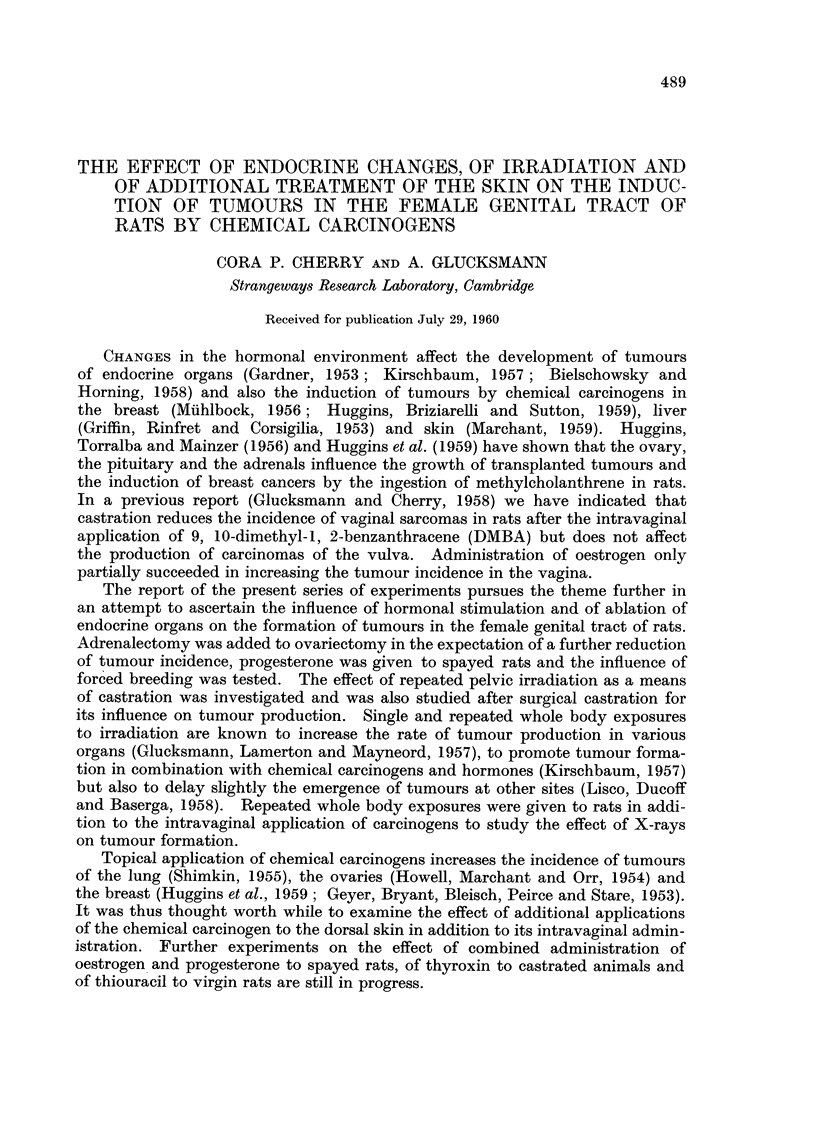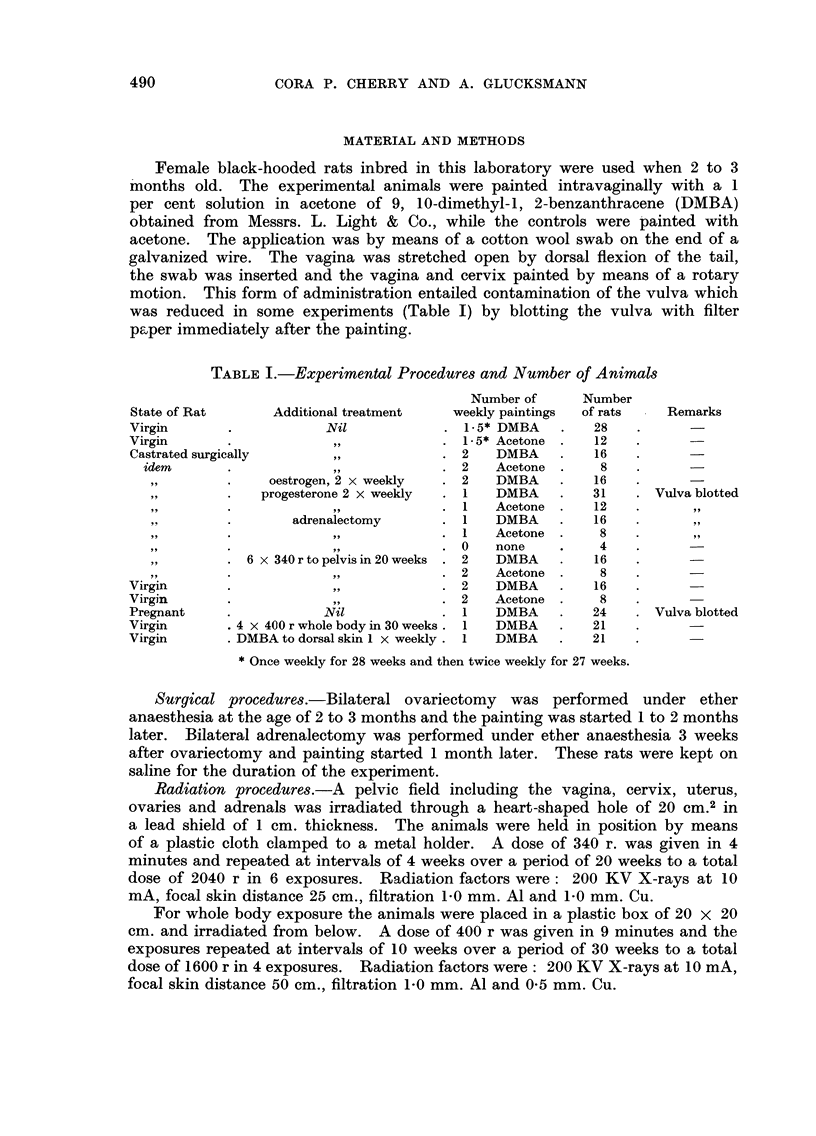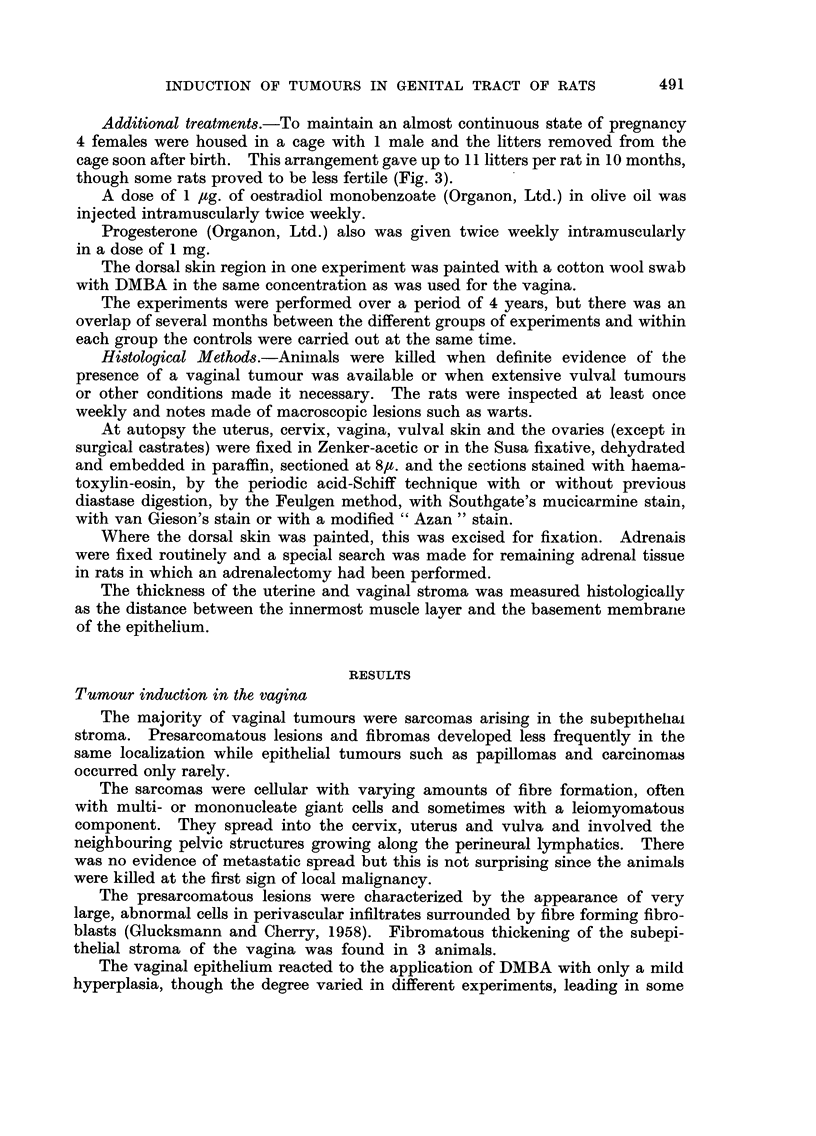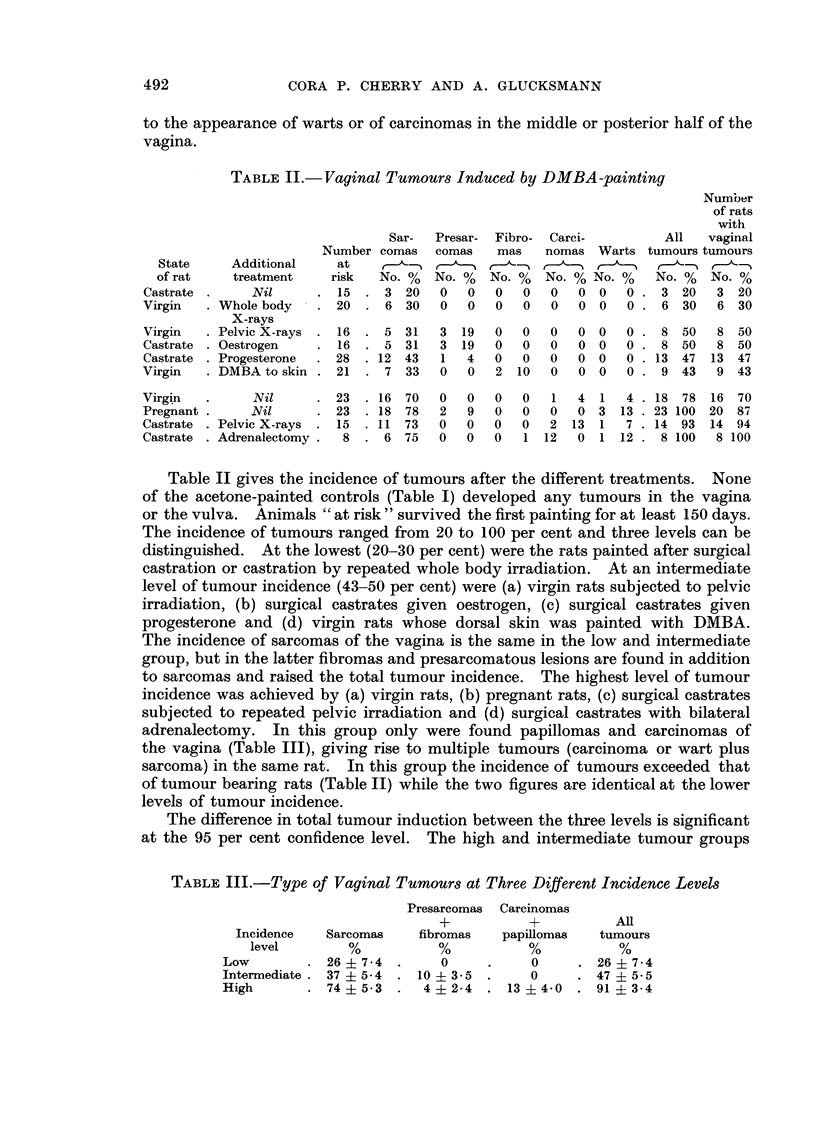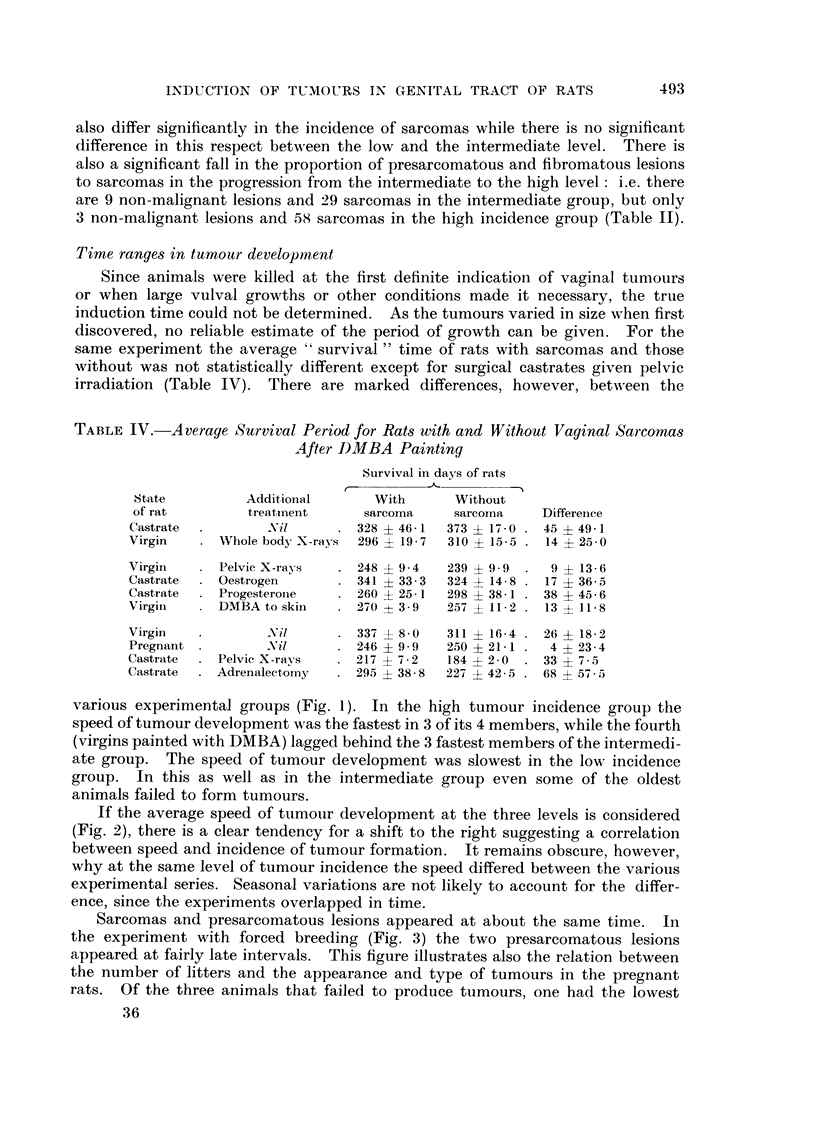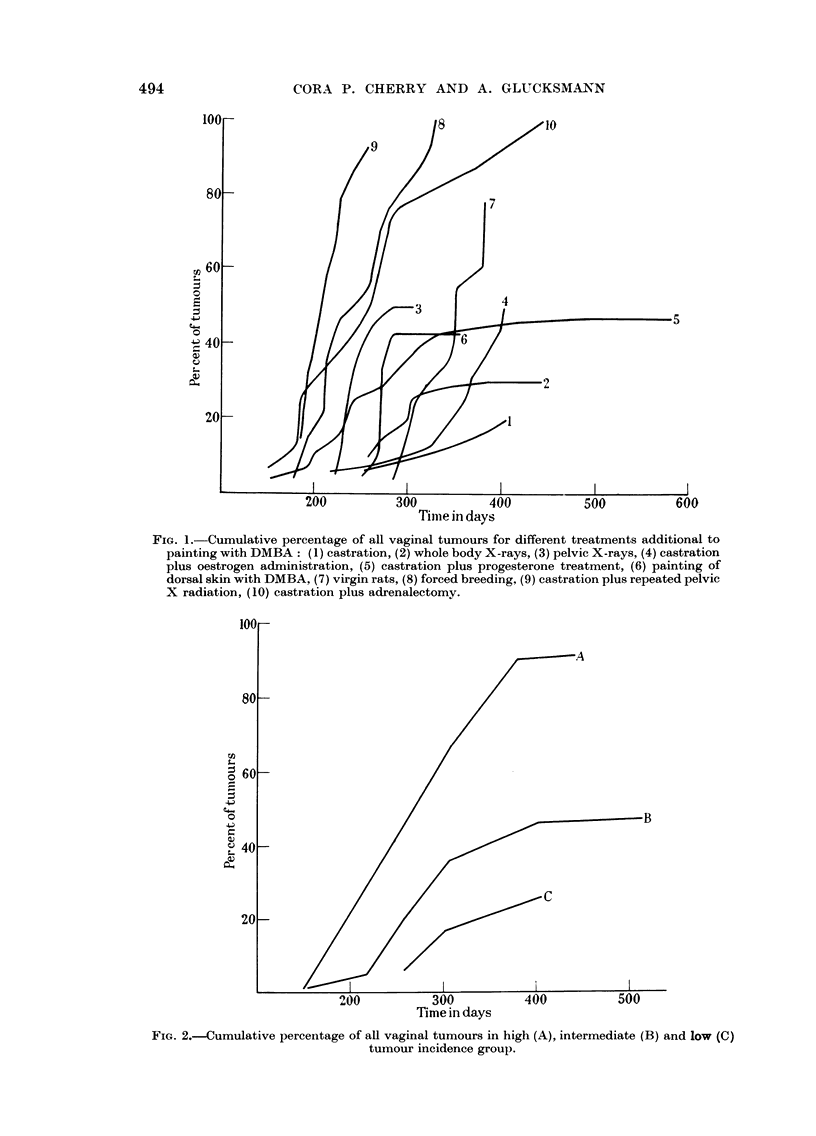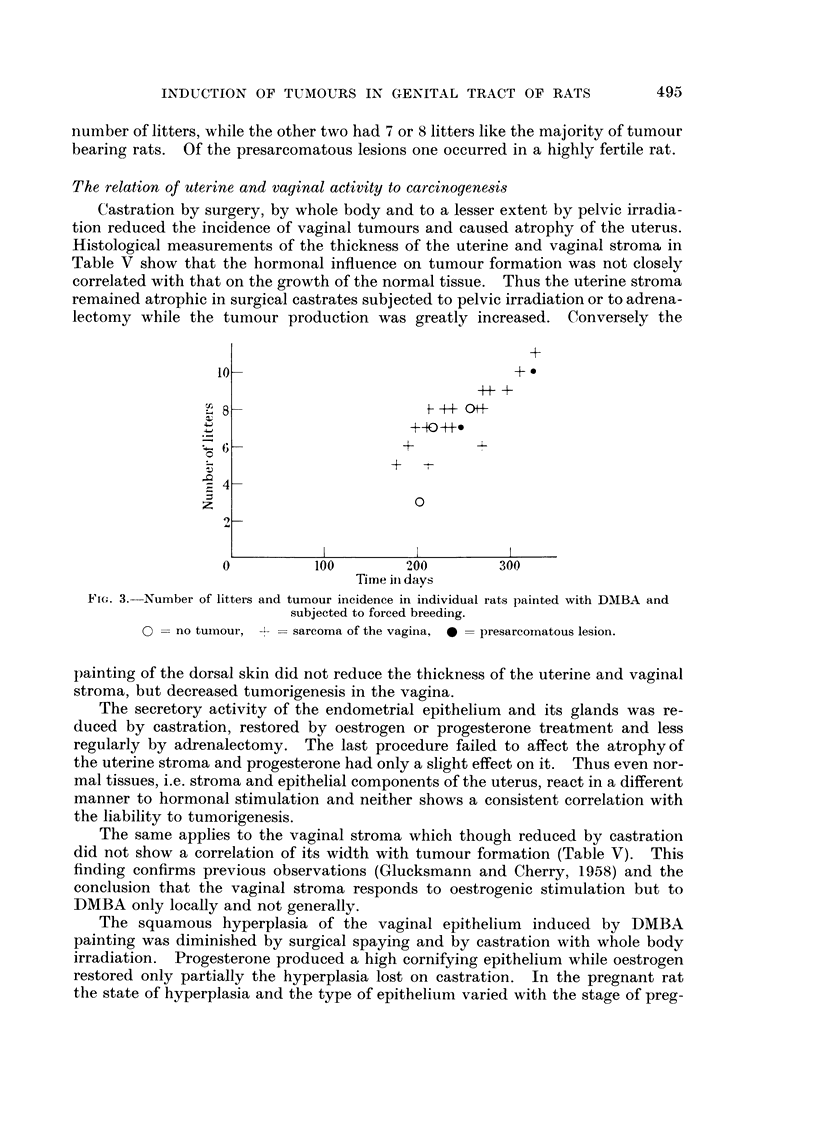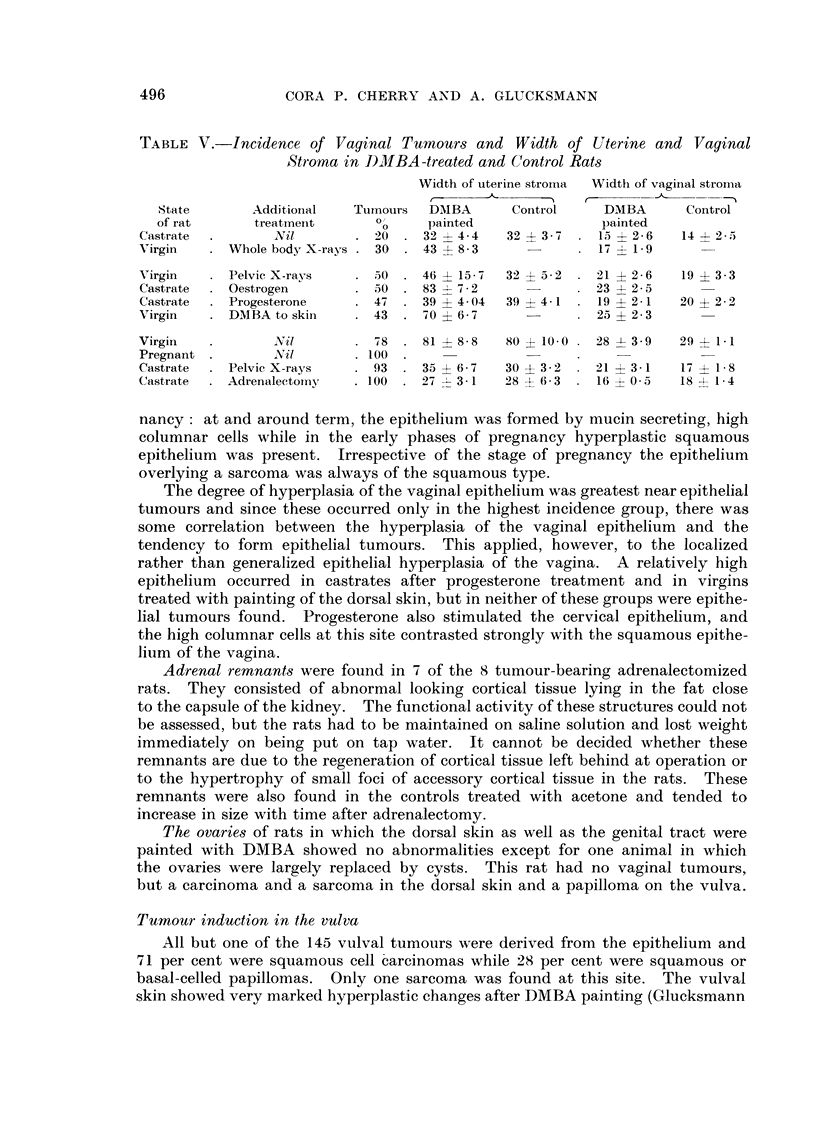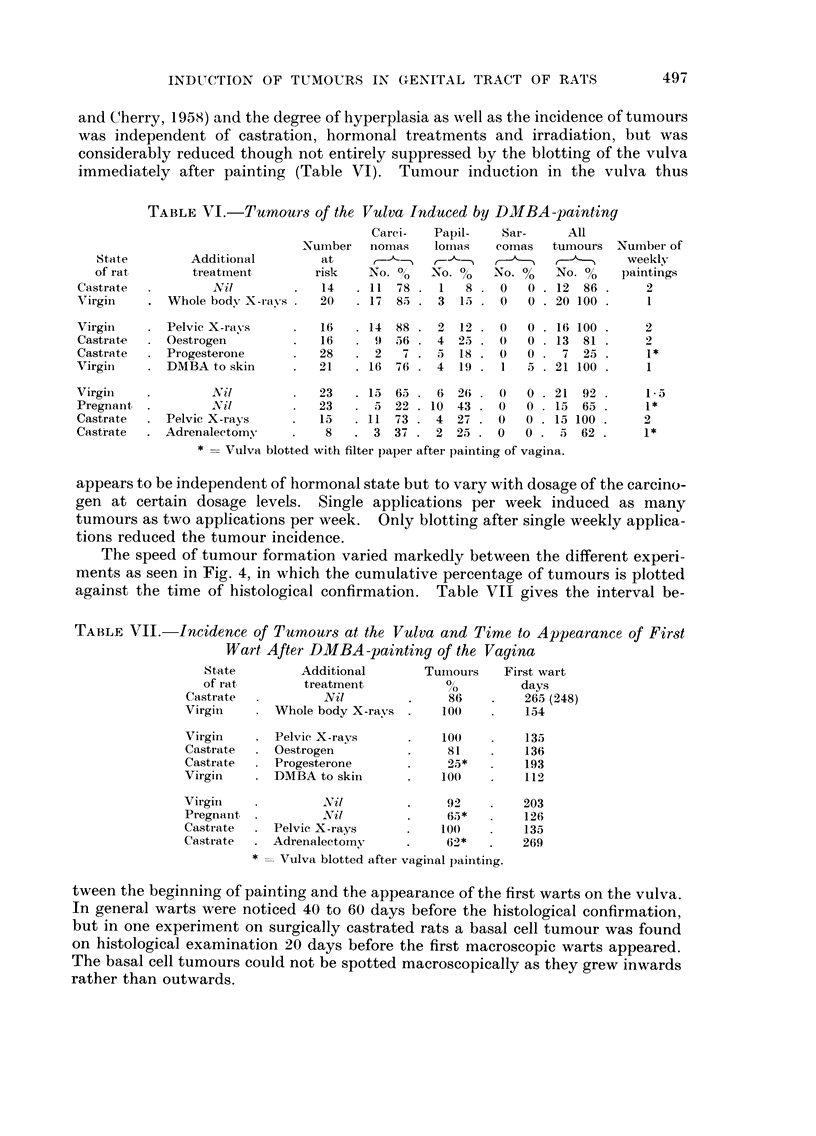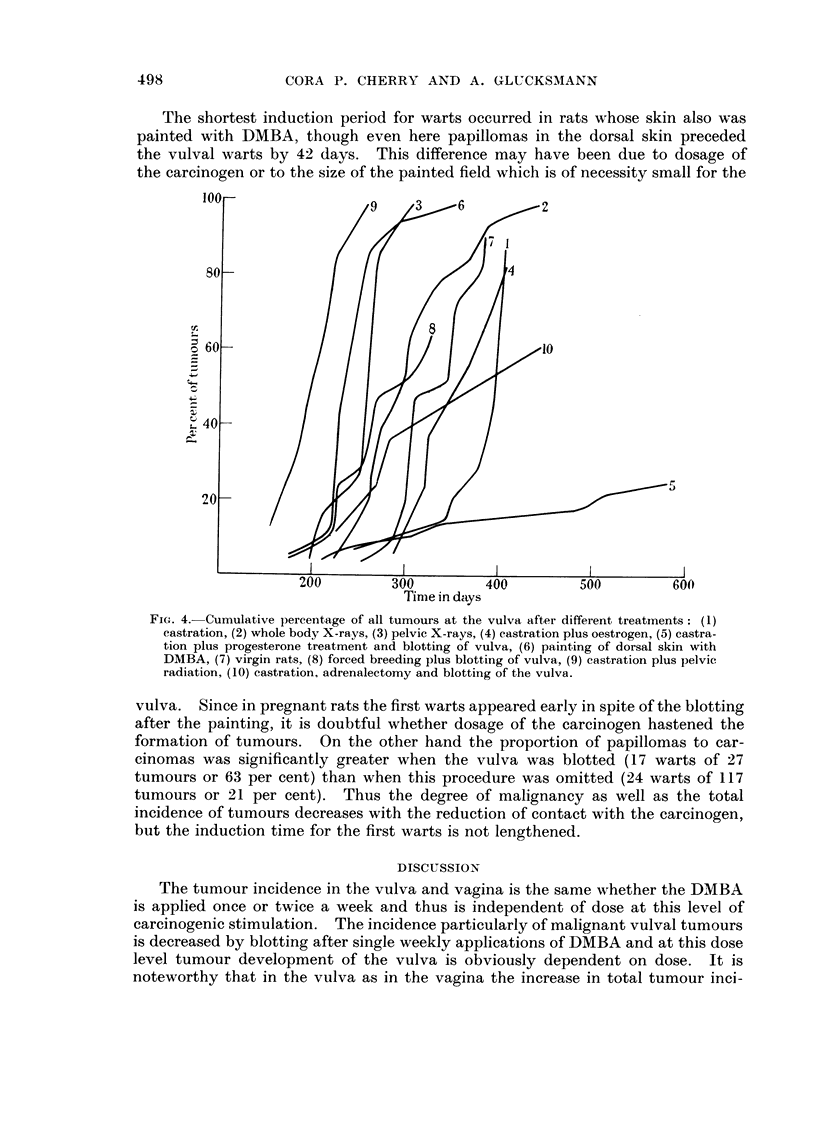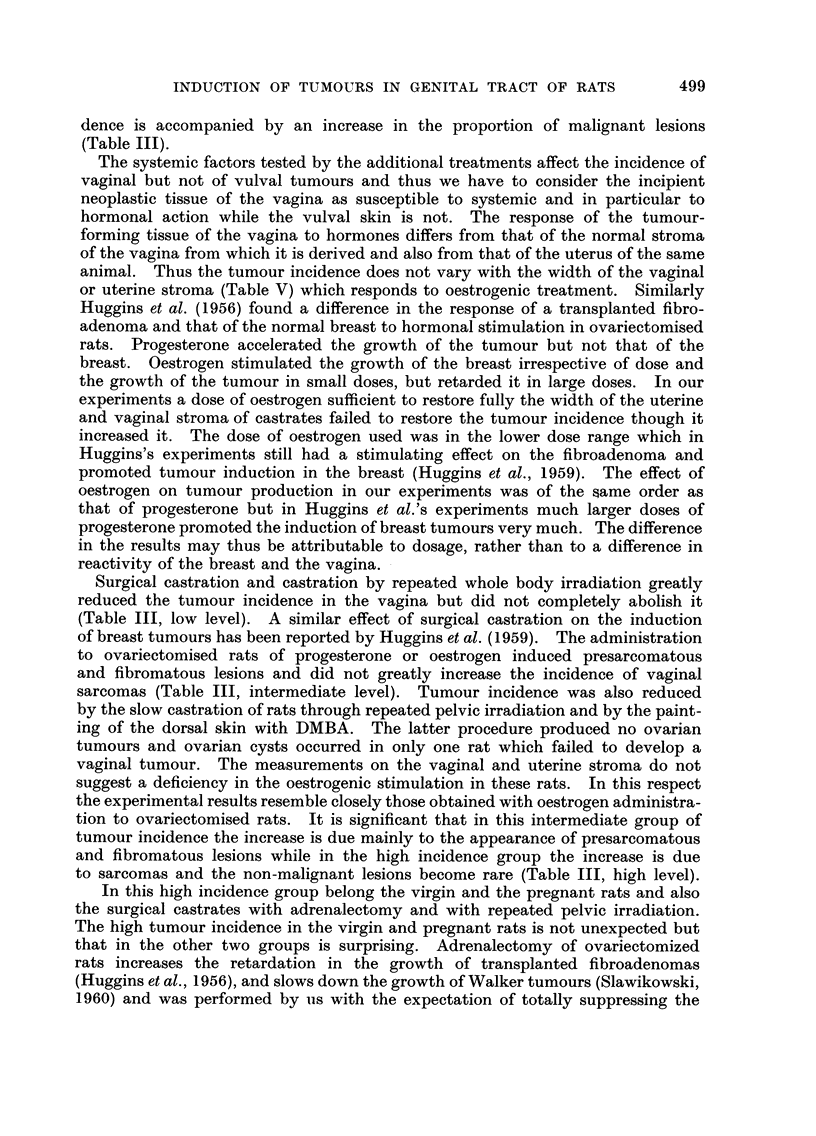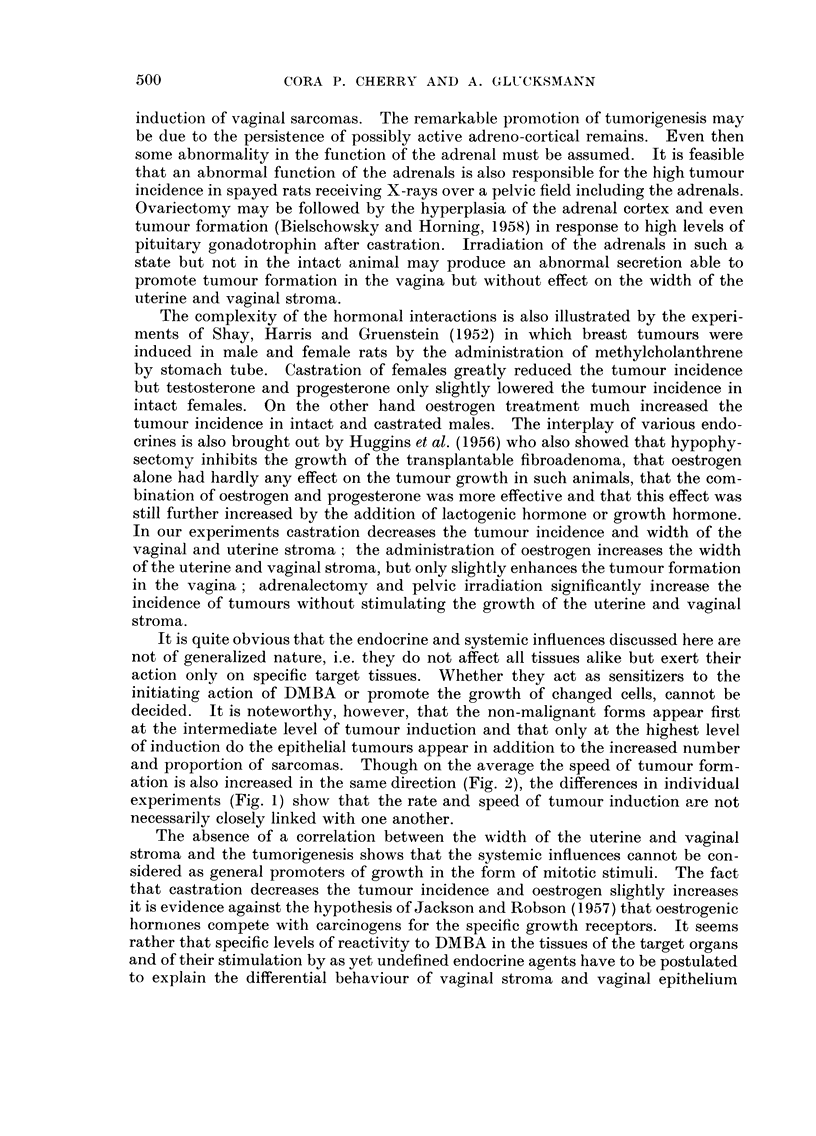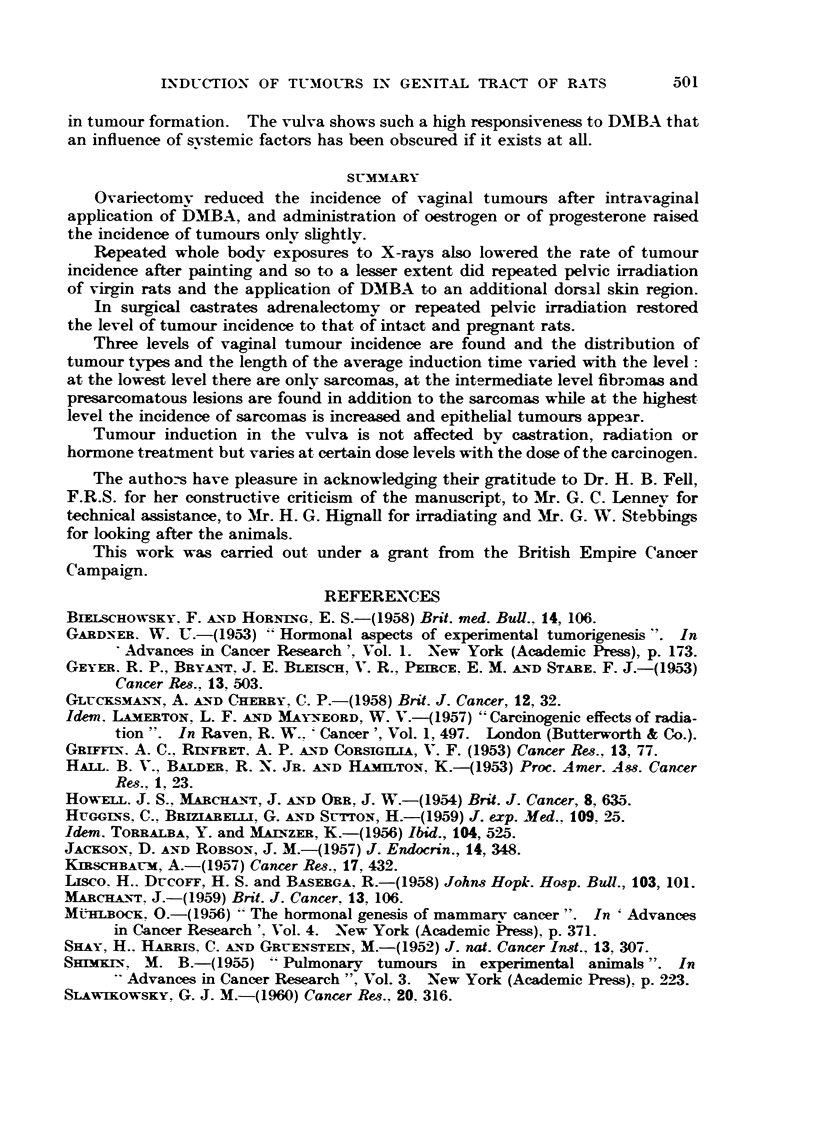# The Effect of Endocrine Changes, of Irradiation and of Additional Treatment of the Skin on the Induction of Tumours in the Female Genital Tract of Rats by Chemical Carcinogens

**DOI:** 10.1038/bjc.1960.54

**Published:** 1960-09

**Authors:** Cora P. Cherry, A. Glucksmann


### THE EFFECT OF ENDOCRINE CHANGES, OF IRRADIATION AND OF ADDITIONAL TREATMENT OF THE SKIN ON THE INDUCTION OF TUMOURS IN THE FEMALE GENITAL TRACT OF RATS BY CHEMICAL CARCINOGENS

CORA P. CHERRY AND A. GLUCKSMANN

*Strangeways Research Laboratory, Cambridge*

Received for publication July 29, 1960

CHANGES in the hormonal environment affect the development of tumours of endocrine organs (Gardner, 1953; Kirschbaum, 1957; Bielschowsky and Horning, 1958) and also the induction of tumours by chemical carcinogens in the breast (Mühlbock, 1956; Huggins, Briziarelli and Sutton, 1959), liver (Griffin, Rinfret and Corsigilia, 1953) and skin (Marchant, 1959). Huggins, Torralba and Mainzer (1956) and Huggins *et al.* (1959) have shown that the ovary, the pituitary and the adrenals influence the growth of transplanted tumours and the induction of breast cancers by the ingestion of methylcholanthrene in rats. In a previous report (Glucksmann and Cherry, 1958) we have indicated that castration reduces the incidence of vaginal sarcomas in rats after the intravaginal application of 9, 10-dimethyl-1, 2-benzanthracene (DMBA) but does not affect the production of carcinomas of the vulva. Administration of oestrogen only partially succeeded in increasing the tumour incidence in the vagina.

The report of the present series of experiments pursues the theme further in an attempt to ascertain the influence of hormonal stimulation and of ablation of endocrine organs on the formation of tumours in the female genital tract of rats. Adrenalectomy was added to ovariectomy in the expectation of a further reduction of tumour incidence, progesterone was given to spayed rats and the influence of forced breeding was tested. The effect of repeated pelvic irradiation as a means of castration was investigated and was also studied after surgical castration for its influence on tumour production. Single and repeated whole body exposures to irradiation are known to increase the rate of tumour production in various organs (Glucksmann, Lamerton and Mayneord, 1957), to promote tumour formation in combination with chemical carcinogens and hormones (Kirschbaum, 1957) but also to delay slightly the emergence of tumours at other sites (Lisco, Ducoff and Baserga, 1958). Repeated whole body exposures were given to rats in addition to the intravaginal application of carcinogens to study the effect of X-rays on tumour formation.

Topical application of chemical carcinogens increases the incidence of tumours of the lung (Shimkin, 1955), the ovaries (Howell, Marchant and Orr, 1954) and the breast (Huggins *et al.*, 1959; Geyer, Bryant, Bleisch, Peirce and Stare, 1953). It was thus thought worth while to examine the effect of additional applications of the chemical carcinogen to the dorsal skin in addition to its intravaginal administration. Further experiments on the effect of combined administration of oestrogen and progesterone to spayed rats, of thyroxin to castrated animals and of thiouracil to virgin rats are still in progress.

### MATERIAL AND METHODS

Female black-hooded rats inbred in this laboratory were used when 2 to 3 months old. The experimental animals were painted intravaginally with a 1 per cent solution in acetone of 9, 10-dimethyl-1, 2-benzanthracene (DMBA) obtained from Messrs. L. Light & Co., while the controls were painted with acetone. The application was by means of a cotton wool swab on the end of a galvanized wire. The vagina was stretched open by dorsal flexion of the tail, the swab was inserted and the vagina and cervix painted by means of a rotary motion. This form of administration entailed contamination of the vulva which was reduced in some experiments (Table I) by blotting the vulva with filter paper immediately after the painting.

TABLE I.—*Experimental Procedures and Number of Animals*

| State of Rat | Additional treatment | Number of weekly paintings | | Number of rats | Remarks |
|---|---|---|---|---|---|
| Virgin | *Nil* | 1·5* | DMBA | 28 | — |
| Virgin | ,, | 1·5* | Acetone | 12 | — |
| Castrated surgically | ,, | 2 | DMBA | 16 | — |
| *idem* | ,, | 2 | Acetone | 8 | — |
| ,, | oestrogen, 2 × weekly | 2 | DMBA | 16 | — |
| ,, | progesterone 2 × weekly | 1 | DMBA | 31 | Vulva blotted |
| ,, | ,, | 1 | Acetone | 12 | ,, |
| ,, | adrenalectomy | 1 | DMBA | 16 | ,, |
| ,, | ,, | 1 | Acetone | 8 | ,, |
| ,, | ,, | 0 | none | 4 | — |
| ,, | 6 × 340 r to pelvis in 20 weeks | 2 | DMBA | 16 | — |
| ,, | ,, | 2 | Acetone | 8 | — |
| Virgin | ,, | 2 | DMBA | 16 | — |
| Virgin | ,, | 2 | Acetone | 8 | — |
| Pregnant | *Nil* | 1 | DMBA | 24 | Vulva blotted |
| Virgin | 4 × 400 r whole body in 30 weeks | 1 | DMBA | 21 | — |
| Virgin | DMBA to dorsal skin 1 × weekly | 1 | DMBA | 21 | — |

* Once weekly for 28 weeks and then twice weekly for 27 weeks.

*Surgical procedures.*—Bilateral ovariectomy was performed under ether anaesthesia at the age of 2 to 3 months and the painting was started 1 to 2 months later. Bilateral adrenalectomy was performed under ether anaesthesia 3 weeks after ovariectomy and painting started 1 month later. These rats were kept on saline for the duration of the experiment.

*Radiation procedures.*—A pelvic field including the vagina, cervix, uterus, ovaries and adrenals was irradiated through a heart-shaped hole of 20 cm.$^2$ in a lead shield of 1 cm. thickness. The animals were held in position by means of a plastic cloth clamped to a metal holder. A dose of 340 r. was given in 4 minutes and repeated at intervals of 4 weeks over a period of 20 weeks to a total dose of 2040 r in 6 exposures. Radiation factors were : 200 KV X-rays at 10 mA, focal skin distance 25 cm., filtration 1·0 mm. Al and 1·0 mm. Cu.

For whole body exposure the animals were placed in a plastic box of 20 × 20 cm. and irradiated from below. A dose of 400 r was given in 9 minutes and the exposures repeated at intervals of 10 weeks over a period of 30 weeks to a total dose of 1600 r in 4 exposures. Radiation factors were : 200 KV X-rays at 10 mA, focal skin distance 50 cm., filtration 1·0 mm. Al and 0·5 mm. Cu.

*Additional treatments.*—To maintain an almost continuous state of pregnancy 4 females were housed in a cage with 1 male and the litters removed from the cage soon after birth. This arrangement gave up to 11 litters per rat in 10 months, though some rats proved to be less fertile (Fig. 3).

A dose of 1 $\mu$g. of oestradiol monobenzoate (Organon, Ltd.) in olive oil was injected intramuscularly twice weekly.

Progesterone (Organon, Ltd.) also was given twice weekly intramuscularly in a dose of 1 mg.

The dorsal skin region in one experiment was painted with a cotton wool swab with DMBA in the same concentration as was used for the vagina.

The experiments were performed over a period of 4 years, but there was an overlap of several months between the different groups of experiments and within each group the controls were carried out at the same time.

*Histological Methods.*—Animals were killed when definite evidence of the presence of a vaginal tumour was available or when extensive vulval tumours or other conditions made it necessary. The rats were inspected at least once weekly and notes made of macroscopic lesions such as warts.

At autopsy the uterus, cervix, vagina, vulval skin and the ovaries (except in surgical castrates) were fixed in Zenker-acetic or in the Susa fixative, dehydrated and embedded in paraffin, sectioned at 8$\mu$. and the sections stained with haematoxylin-eosin, by the periodic acid-Schiff technique with or without previous diastase digestion, by the Feulgen method, with Southgate's mucicarmine stain, with van Gieson's stain or with a modified " Azan " stain.

Where the dorsal skin was painted, this was excised for fixation. Adrenals were fixed routinely and a special search was made for remaining adrenal tissue in rats in which an adrenalectomy had been performed.

The thickness of the uterine and vaginal stroma was measured histologically as the distance between the innermost muscle layer and the basement membrane of the epithelium.

### RESULTS

### *Tumour induction in the vagina*

The majority of vaginal tumours were sarcomas arising in the subepithelial stroma. Presarcomatous lesions and fibromas developed less frequently in the same localization while epithelial tumours such as papillomas and carcinomas occurred only rarely.

The sarcomas were cellular with varying amounts of fibre formation, often with multi- or mononucleate giant cells and sometimes with a leiomyomatous component. They spread into the cervix, uterus and vulva and involved the neighbouring pelvic structures growing along the perineural lymphatics. There was no evidence of metastatic spread but this is not surprising since the animals were killed at the first sign of local malignancy.

The presarcomatous lesions were characterized by the appearance of very large, abnormal cells in perivascular infiltrates surrounded by fibre forming fibroblasts (Glucksmann and Cherry, 1958). Fibromatous thickening of the subepithelial stroma of the vagina was found in 3 animals.

The vaginal epithelium reacted to the application of DMBA with only a mild hyperplasia, though the degree varied in different experiments, leading in some to the appearance of warts or of carcinomas in the middle or posterior half of the vagina.

TABLE II.—*Vaginal Tumours Induced by DMBA-painting*

| State of rat | Additional treatment | Number at risk | Sarcomas No. | Sarcomas % | Presarcomas No. | Presarcomas % | Fibromas No. | Fibromas % | Carcinomas No. | Carcinomas % | Warts No. | Warts % | All tumours No. | All tumours % | Number of rats with vaginal tumours No. | Number of rats with vaginal tumours % |
|---|---|---|---|---|---|---|---|---|---|---|---|---|---|---|---|---|
| Castrate | *Nil* | 15 | 3 | 20 | 0 | 0 | 0 | 0 | 0 | 0 | 0 | 0 | 3 | 20 | 3 | 20 |
| Virgin | Whole body X-rays | 20 | 6 | 30 | 0 | 0 | 0 | 0 | 0 | 0 | 0 | 0 | 6 | 30 | 6 | 30 |
| Virgin | Pelvic X-rays | 16 | 5 | 31 | 3 | 19 | 0 | 0 | 0 | 0 | 0 | 0 | 8 | 50 | 8 | 50 |
| Castrate | Oestrogen | 16 | 5 | 31 | 3 | 19 | 0 | 0 | 0 | 0 | 0 | 0 | 8 | 50 | 8 | 50 |
| Castrate | Progesterone | 28 | 12 | 43 | 1 | 4 | 0 | 0 | 0 | 0 | 0 | 0 | 13 | 47 | 13 | 47 |
| Virgin | DMBA to skin | 21 | 7 | 33 | 0 | 0 | 2 | 10 | 0 | 0 | 0 | 0 | 9 | 43 | 9 | 43 |
| Virgin | *Nil* | 23 | 16 | 70 | 0 | 0 | 0 | 0 | 1 | 4 | 1 | 4 | 18 | 78 | 16 | 70 |
| Pregnant | *Nil* | 23 | 18 | 78 | 2 | 9 | 0 | 0 | 0 | 0 | 3 | 13 | 23 | 100 | 20 | 87 |
| Castrate | Pelvic X-rays | 15 | 11 | 73 | 0 | 0 | 0 | 0 | 2 | 13 | 1 | 7 | 14 | 93 | 14 | 94 |
| Castrate | Adrenalectomy | 8 | 6 | 75 | 0 | 0 | 0 | 1 | 12 | 0 | 1 | 12 | 8 | 100 | 8 | 100 |

Table II gives the incidence of tumours after the different treatments. None of the acetone-painted controls (Table I) developed any tumours in the vagina or the vulva. Animals "at risk" survived the first painting for at least 150 days. The incidence of tumours ranged from 20 to 100 per cent and three levels can be distinguished. At the lowest (20–30 per cent) were the rats painted after surgical castration or castration by repeated whole body irradiation. At an intermediate level of tumour incidence (43–50 per cent) were (a) virgin rats subjected to pelvic irradiation, (b) surgical castrates given oestrogen, (c) surgical castrates given progesterone and (d) virgin rats whose dorsal skin was painted with DMBA. The incidence of sarcomas of the vagina is the same in the low and intermediate group, but in the latter fibromas and presarcomatous lesions are found in addition to sarcomas and raised the total tumour incidence. The highest level of tumour incidence was achieved by (a) virgin rats, (b) pregnant rats, (c) surgical castrates subjected to repeated pelvic irradiation and (d) surgical castrates with bilateral adrenalectomy. In this group only were found papillomas and carcinomas of the vagina (Table III), giving rise to multiple tumours (carcinoma or wart plus sarcoma) in the same rat. In this group the incidence of tumours exceeded that of tumour bearing rats (Table II) while the two figures are identical at the lower levels of tumour incidence.

The difference in total tumour induction between the three levels is significant at the 95 per cent confidence level. The high and intermediate tumour groups

TABLE III.—*Type of Vaginal Tumours at Three Different Incidence Levels*

| Incidence level | Sarcomas % | Presarcomas + fibromas % | Carcinomas + papillomas % | All tumours % |
|---|---|---|---|---|
| Low | $26 \pm 7 \cdot 4$ | 0 | 0 | $26 \pm 7 \cdot 4$ |
| Intermediate | $37 \pm 5 \cdot 4$ | $10 \pm 3 \cdot 5$ | 0 | $47 \pm 5 \cdot 5$ |
| High | $74 \pm 5 \cdot 3$ | $4 \pm 2 \cdot 4$ | $13 \pm 4 \cdot 0$ | $91 \pm 3 \cdot 4$ |

also differ significantly in the incidence of sarcomas while there is no significant difference in this respect between the low and the intermediate level. There is also a significant fall in the proportion of presarcomatous and fibromatous lesions to sarcomas in the progression from the intermediate to the high level : i.e. there are 9 non-malignant lesions and 29 sarcomas in the intermediate group, but only 3 non-malignant lesions and 58 sarcomas in the high incidence group (Table II).

*Time ranges in tumour development*

Since animals were killed at the first definite indication of vaginal tumours or when large vulval growths or other conditions made it necessary, the true induction time could not be determined. As the tumours varied in size when first discovered, no reliable estimate of the period of growth can be given. For the same experiment the average " survival " time of rats with sarcomas and those without was not statistically different except for surgical castrates given pelvic irradiation (Table IV). There are marked differences, however, between the

TABLE IV.—*Average Survival Period for Rats with and Without Vaginal Sarcomas After DMBA Painting*

| State of rat | Additional treatment | Survival in days of rats: With sarcoma | Survival in days of rats: Without sarcoma | Difference |
|---|---|---|---|---|
| Castrate | *Nil* | $328 \pm 46 \cdot 1$ | $373 \pm 17 \cdot 0$ | $45 \pm 49 \cdot 1$ |
| Virgin | Whole body X-rays | $296 \pm 19 \cdot 7$ | $310 \pm 15 \cdot 5$ | $14 \pm 25 \cdot 0$ |
| Virgin | Pelvic X-rays | $248 \pm 9 \cdot 4$ | $239 \pm 9 \cdot 9$ | $9 \pm 13 \cdot 6$ |
| Castrate | Oestrogen | $341 \pm 33 \cdot 3$ | $324 \pm 14 \cdot 8$ | $17 \pm 36 \cdot 5$ |
| Castrate | Progesterone | $260 \pm 25 \cdot 1$ | $298 \pm 38 \cdot 1$ | $38 \pm 45 \cdot 6$ |
| Virgin | DMBA to skin | $270 \pm 3 \cdot 9$ | $257 \pm 11 \cdot 2$ | $13 \pm 11 \cdot 8$ |
| Virgin | *Nil* | $337 \pm 8 \cdot 0$ | $311 \pm 16 \cdot 4$ | $26 \pm 18 \cdot 2$ |
| Pregnant | *Nil* | $246 \pm 9 \cdot 9$ | $250 \pm 21 \cdot 1$ | $4 \pm 23 \cdot 4$ |
| Castrate | Pelvic X-rays | $217 \pm 7 \cdot 2$ | $184 \pm 2 \cdot 0$ | $33 \pm 7 \cdot 5$ |
| Castrate | Adrenalectomy | $295 \pm 38 \cdot 8$ | $227 \pm 42 \cdot 5$ | $68 \pm 57 \cdot 5$ |

various experimental groups (Fig. 1). In the high tumour incidence group the speed of tumour development was the fastest in 3 of its 4 members, while the fourth (virgins painted with DMBA) lagged behind the 3 fastest members of the intermediate group. The speed of tumour development was slowest in the low incidence group. In this as well as in the intermediate group even some of the oldest animals failed to form tumours.

If the average speed of tumour development at the three levels is considered (Fig. 2), there is a clear tendency for a shift to the right suggesting a correlation between speed and incidence of tumour formation. It remains obscure, however, why at the same level of tumour incidence the speed differed between the various experimental series. Seasonal variations are not likely to account for the difference, since the experiments overlapped in time.

Sarcomas and presarcomatous lesions appeared at about the same time. In the experiment with forced breeding (Fig. 3) the two presarcomatous lesions appeared at fairly late intervals. This figure illustrates also the relation between the number of litters and the appearance and type of tumours in the pregnant rats. Of the three animals that failed to produce tumours, one had the lowest

FIG. 1.—Cumulative percentage of all vaginal tumours for different treatments additional to painting with DMBA : (1) castration, (2) whole body X-rays, (3) pelvic X-rays, (4) castration plus oestrogen administration, (5) castration plus progesterone treatment, (6) painting of dorsal skin with DMBA, (7) virgin rats, (8) forced breeding, (9) castration plus repeated pelvic X radiation, (10) castration plus adrenalectomy.

FIG. 2.—Cumulative percentage of all vaginal tumours in high (A), intermediate (B) and low (C) tumour incidence group.

number of litters, while the other two had 7 or 8 litters like the majority of tumour bearing rats. Of the presarcomatous lesions one occurred in a highly fertile rat.

*The relation of uterine and vaginal activity to carcinogenesis*

Castration by surgery, by whole body and to a lesser extent by pelvic irradiation reduced the incidence of vaginal tumours and caused atrophy of the uterus. Histological measurements of the thickness of the uterine and vaginal stroma in Table V show that the hormonal influence on tumour formation was not closely correlated with that on the growth of the normal tissue. Thus the uterine stroma remained atrophic in surgical castrates subjected to pelvic irradiation or to adrenalectomy while the tumour production was greatly increased. Conversely the

FIG. 3.—Number of litters and tumour incidence in individual rats painted with DMBA and subjected to forced breeding.

○ = no tumour, + = sarcoma of the vagina, ● = presarcomatous lesion.

painting of the dorsal skin did not reduce the thickness of the uterine and vaginal stroma, but decreased tumorigenesis in the vagina.

The secretory activity of the endometrial epithelium and its glands was reduced by castration, restored by oestrogen or progesterone treatment and less regularly by adrenalectomy. The last procedure failed to affect the atrophy of the uterine stroma and progesterone had only a slight effect on it. Thus even normal tissues, i.e. stroma and epithelial components of the uterus, react in a different manner to hormonal stimulation and neither shows a consistent correlation with the liability to tumorigenesis.

The same applies to the vaginal stroma which though reduced by castration did not show a correlation of its width with tumour formation (Table V). This finding confirms previous observations (Glucksmann and Cherry, 1958) and the conclusion that the vaginal stroma responds to oestrogenic stimulation but to DMBA only locally and not generally.

The squamous hyperplasia of the vaginal epithelium induced by DMBA painting was diminished by surgical spaying and by castration with whole body irradiation. Progesterone produced a high cornifying epithelium while oestrogen restored only partially the hyperplasia lost on castration. In the pregnant rat the state of hyperplasia and the type of epithelium varied with the stage of preg-

TABLE V.—*Incidence of Vaginal Tumours and Width of Uterine and Vaginal Stroma in DMBA-treated and Control Rats*

| | | | Width of uterine stroma | | Width of vaginal stroma | |
|---|---|---|---|---|---|---|
| State of rat | Additional treatment | Tumours % | DMBA painted | Control | DMBA painted | Control |
| Castrate | *Nil* | 20 | 32 ± 4·4 | 32 ± 3·7 | 15 ± 2·6 | 14 ± 2·5 |
| Virgin | Whole body X-rays | 30 | 43 ± 8·3 | — | 17 ± 1·9 | — |
| Virgin | Pelvic X-rays | 50 | 46 ± 15·7 | 32 ± 5·2 | 21 ± 2·6 | 19 ± 3·3 |
| Castrate | Oestrogen | 50 | 83 ± 7·2 | — | 23 ± 2·5 | — |
| Castrate | Progesterone | 47 | 39 ± 4·04 | 39 ± 4·1 | 19 ± 2·1 | 20 ± 2·2 |
| Virgin | DMBA to skin | 43 | 70 ± 6·7 | — | 25 ± 2·3 | — |
| Virgin | *Nil* | 78 | 81 ± 8·8 | 80 ± 10·0 | 28 ± 3·9 | 29 ± 1·1 |
| Pregnant | *Nil* | 100 | — | — | — | — |
| Castrate | Pelvic X-rays | 93 | 35 ± 6·7 | 30 ± 3·2 | 21 ± 3·1 | 17 ± 1·8 |
| Castrate | Adrenalectomy | 100 | 27 ± 3·1 | 28 ± 6·3 | 16 ± 0·5 | 18 ± 1·4 |

nancy : at and around term, the epithelium was formed by mucin secreting, high columnar cells while in the early phases of pregnancy hyperplastic squamous epithelium was present. Irrespective of the stage of pregnancy the epithelium overlying a sarcoma was always of the squamous type.

The degree of hyperplasia of the vaginal epithelium was greatest near epithelial tumours and since these occurred only in the highest incidence group, there was some correlation between the hyperplasia of the vaginal epithelium and the tendency to form epithelial tumours. This applied, however, to the localized rather than generalized epithelial hyperplasia of the vagina. A relatively high epithelium occurred in castrates after progesterone treatment and in virgins treated with painting of the dorsal skin, but in neither of these groups were epithelial tumours found. Progesterone also stimulated the cervical epithelium, and the high columnar cells at this site contrasted strongly with the squamous epithelium of the vagina.

*Adrenal remnants* were found in 7 of the 8 tumour-bearing adrenalectomized rats. They consisted of abnormal looking cortical tissue lying in the fat close to the capsule of the kidney. The functional activity of these structures could not be assessed, but the rats had to be maintained on saline solution and lost weight immediately on being put on tap water. It cannot be decided whether these remnants are due to the regeneration of cortical tissue left behind at operation or to the hypertrophy of small foci of accessory cortical tissue in the rats. These remnants were also found in the controls treated with acetone and tended to increase in size with time after adrenalectomy.

*The ovaries* of rats in which the dorsal skin as well as the genital tract were painted with DMBA showed no abnormalities except for one animal in which the ovaries were largely replaced by cysts. This rat had no vaginal tumours, but a carcinoma and a sarcoma in the dorsal skin and a papilloma on the vulva.

*Tumour induction in the vulva*

All but one of the 145 vulval tumours were derived from the epithelium and 71 per cent were squamous cell carcinomas while 28 per cent were squamous or basal-celled papillomas. Only one sarcoma was found at this site. The vulval skin showed very marked hyperplastic changes after DMBA painting (Glucksmann and Cherry, 1958) and the degree of hyperplasia as well as the incidence of tumours was independent of castration, hormonal treatments and irradiation, but was considerably reduced though not entirely suppressed by the blotting of the vulva immediately after painting (Table VI). Tumour induction in the vulva thus

TABLE VI.—*Tumours of the Vulva Induced by DMBA-painting*

| State of rat | Additional treatment | Number at risk | Carcinomas No. | Carcinomas % | Papillomas No. | Papillomas % | Sarcomas No. | Sarcomas % | All tumours No. | All tumours % | Number of weekly paintings |
|---|---|---|---|---|---|---|---|---|---|---|---|
| Castrate | *Nil* | 14 | 11 | 78 | 1 | 8 | 0 | 0 | 12 | 86 | 2 |
| Virgin | Whole body X-rays | 20 | 17 | 85 | 3 | 15 | 0 | 0 | 20 | 100 | 1 |
| Virgin | Pelvic X-rays | 16 | 14 | 88 | 2 | 12 | 0 | 0 | 16 | 100 | 2 |
| Castrate | Oestrogen | 16 | 9 | 56 | 4 | 25 | 0 | 0 | 13 | 81 | 2 |
| Castrate | Progesterone | 28 | 2 | 7 | 5 | 18 | 0 | 0 | 7 | 25 | 1* |
| Virgin | DMBA to skin | 21 | 16 | 76 | 4 | 19 | 1 | 5 | 21 | 100 | 1 |
| Virgin | *Nil* | 23 | 15 | 65 | 6 | 26 | 0 | 0 | 21 | 92 | 1·5 |
| Pregnant | *Nil* | 23 | 5 | 22 | 10 | 43 | 0 | 0 | 15 | 65 | 1* |
| Castrate | Pelvic X-rays | 15 | 11 | 73 | 4 | 27 | 0 | 0 | 15 | 100 | 2 |
| Castrate | Adrenalectomy | 8 | 3 | 37 | 2 | 25 | 0 | 0 | 5 | 62 | 1* |

* = Vulva blotted with filter paper after painting of vagina.

appears to be independent of hormonal state but to vary with dosage of the carcinogen at certain dosage levels. Single applications per week induced as many tumours as two applications per week. Only blotting after single weekly applications reduced the tumour incidence.

The speed of tumour formation varied markedly between the different experiments as seen in Fig. 4, in which the cumulative percentage of tumours is plotted against the time of histological confirmation. Table VII gives the interval be-

TABLE VII.—*Incidence of Tumours at the Vulva and Time to Appearance of First Wart After DMBA-painting of the Vagina*

| State of rat | Additional treatment | Tumours % | First wart days |
|---|---|---|---|
| Castrate | *Nil* | 86 | 265 (248) |
| Virgin | Whole body X-rays | 100 | 154 |
| Virgin | Pelvic X-rays | 100 | 135 |
| Castrate | Oestrogen | 81 | 136 |
| Castrate | Progesterone | 25* | 193 |
| Virgin | DMBA to skin | 100 | 112 |
| Virgin | *Nil* | 92 | 203 |
| Pregnant | *Nil* | 65* | 126 |
| Castrate | Pelvic X-rays | 100 | 135 |
| Castrate | Adrenalectomy | 62* | 269 |

* = Vulva blotted after vaginal painting.

tween the beginning of painting and the appearance of the first warts on the vulva. In general warts were noticed 40 to 60 days before the histological confirmation, but in one experiment on surgically castrated rats a basal cell tumour was found on histological examination 20 days before the first macroscopic warts appeared. The basal cell tumours could not be spotted macroscopically as they grew inwards rather than outwards.

The shortest induction period for warts occurred in rats whose skin also was painted with DMBA, though even here papillomas in the dorsal skin preceded the vulval warts by 42 days. This difference may have been due to dosage of the carcinogen or to the size of the painted field which is of necessity small for the

FIG. 4.—Cumulative percentage of all tumours at the vulva after different treatments: (1) castration, (2) whole body X-rays, (3) pelvic X-rays, (4) castration plus oestrogen, (5) castration plus progesterone treatment and blotting of vulva, (6) painting of dorsal skin with DMBA, (7) virgin rats, (8) forced breeding plus blotting of vulva, (9) castration plus pelvic radiation, (10) castration, adrenalectomy and blotting of the vulva.

vulva. Since in pregnant rats the first warts appeared early in spite of the blotting after the painting, it is doubtful whether dosage of the carcinogen hastened the formation of tumours. On the other hand the proportion of papillomas to carcinomas was significantly greater when the vulva was blotted (17 warts of 27 tumours or 63 per cent) than when this procedure was omitted (24 warts of 117 tumours or 21 per cent). Thus the degree of malignancy as well as the total incidence of tumours decreases with the reduction of contact with the carcinogen, but the induction time for the first warts is not lengthened.

### DISCUSSION

The tumour incidence in the vulva and vagina is the same whether the DMBA is applied once or twice a week and thus is independent of dose at this level of carcinogenic stimulation. The incidence particularly of malignant vulval tumours is decreased by blotting after single weekly applications of DMBA and at this dose level tumour development of the vulva is obviously dependent on dose. It is noteworthy that in the vulva as in the vagina the increase in total tumour incidence is accompanied by an increase in the proportion of malignant lesions (Table III).

The systemic factors tested by the additional treatments affect the incidence of vaginal but not of vulval tumours and thus we have to consider the incipient neoplastic tissue of the vagina as susceptible to systemic and in particular to hormonal action while the vulval skin is not. The response of the tumour-forming tissue of the vagina to hormones differs from that of the normal stroma of the vagina from which it is derived and also from that of the uterus of the same animal. Thus the tumour incidence does not vary with the width of the vaginal or uterine stroma (Table V) which responds to oestrogenic treatment. Similarly Huggins *et al.* (1956) found a difference in the response of a transplanted fibro-adenoma and that of the normal breast to hormonal stimulation in ovariectomised rats. Progesterone accelerated the growth of the tumour but not that of the breast. Oestrogen stimulated the growth of the breast irrespective of dose and the growth of the tumour in small doses, but retarded it in large doses. In our experiments a dose of oestrogen sufficient to restore fully the width of the uterine and vaginal stroma of castrates failed to restore the tumour incidence though it increased it. The dose of oestrogen used was in the lower dose range which in Huggins's experiments still had a stimulating effect on the fibroadenoma and promoted tumour induction in the breast (Huggins *et al.*, 1959). The effect of oestrogen on tumour production in our experiments was of the same order as that of progesterone but in Huggins *et al.*'s experiments much larger doses of progesterone promoted the induction of breast tumours very much. The difference in the results may thus be attributable to dosage, rather than to a difference in reactivity of the breast and the vagina.

Surgical castration and castration by repeated whole body irradiation greatly reduced the tumour incidence in the vagina but did not completely abolish it (Table III, low level). A similar effect of surgical castration on the induction of breast tumours has been reported by Huggins *et al.* (1959). The administration to ovariectomised rats of progesterone or oestrogen induced presarcomatous and fibromatous lesions and did not greatly increase the incidence of vaginal sarcomas (Table III, intermediate level). Tumour incidence was also reduced by the slow castration of rats through repeated pelvic irradiation and by the painting of the dorsal skin with DMBA. The latter procedure produced no ovarian tumours and ovarian cysts occurred in only one rat which failed to develop a vaginal tumour. The measurements on the vaginal and uterine stroma do not suggest a deficiency in the oestrogenic stimulation in these rats. In this respect the experimental results resemble closely those obtained with oestrogen administration to ovariectomised rats. It is significant that in this intermediate group of tumour incidence the increase is due mainly to the appearance of presarcomatous and fibromatous lesions while in the high incidence group the increase is due to sarcomas and the non-malignant lesions become rare (Table III, high level).

In this high incidence group belong the virgin and the pregnant rats and also the surgical castrates with adrenalectomy and with repeated pelvic irradiation. The high tumour incidence in the virgin and pregnant rats is not unexpected but that in the other two groups is surprising. Adrenalectomy of ovariectomized rats increases the retardation in the growth of transplanted fibroadenomas (Huggins *et al.*, 1956), and slows down the growth of Walker tumours (Slawikowski, 1960) and was performed by us with the expectation of totally suppressing the induction of vaginal sarcomas. The remarkable promotion of tumorigenesis may be due to the persistence of possibly active adreno-cortical remains. Even then some abnormality in the function of the adrenal must be assumed. It is feasible that an abnormal function of the adrenals is also responsible for the high tumour incidence in spayed rats receiving X-rays over a pelvic field including the adrenals. Ovariectomy may be followed by the hyperplasia of the adrenal cortex and even tumour formation (Bielschowsky and Horning, 1958) in response to high levels of pituitary gonadotrophin after castration. Irradiation of the adrenals in such a state but not in the intact animal may produce an abnormal secretion able to promote tumour formation in the vagina but without effect on the width of the uterine and vaginal stroma.

The complexity of the hormonal interactions is also illustrated by the experiments of Shay, Harris and Gruenstein (1952) in which breast tumours were induced in male and female rats by the administration of methylcholanthrene by stomach tube. Castration of females greatly reduced the tumour incidence but testosterone and progesterone only slightly lowered the tumour incidence in intact females. On the other hand oestrogen treatment much increased the tumour incidence in intact and castrated males. The interplay of various endocrines is also brought out by Huggins *et al.* (1956) who also showed that hypophysectomy inhibits the growth of the transplantable fibroadenoma, that oestrogen alone had hardly any effect on the tumour growth in such animals, that the combination of oestrogen and progesterone was more effective and that this effect was still further increased by the addition of lactogenic hormone or growth hormone. In our experiments castration decreases the tumour incidence and width of the vaginal and uterine stroma ; the administration of oestrogen increases the width of the uterine and vaginal stroma, but only slightly enhances the tumour formation in the vagina ; adrenalectomy and pelvic irradiation significantly increase the incidence of tumours without stimulating the growth of the uterine and vaginal stroma.

It is quite obvious that the endocrine and systemic influences discussed here are not of generalized nature, i.e. they do not affect all tissues alike but exert their action only on specific target tissues. Whether they act as sensitizers to the initiating action of DMBA or promote the growth of changed cells, cannot be decided. It is noteworthy, however, that the non-malignant forms appear first at the intermediate level of tumour induction and that only at the highest level of induction do the epithelial tumours appear in addition to the increased number and proportion of sarcomas. Though on the average the speed of tumour formation is also increased in the same direction (Fig. 2), the differences in individual experiments (Fig. 1) show that the rate and speed of tumour induction are not necessarily closely linked with one another.

The absence of a correlation between the width of the uterine and vaginal stroma and the tumorigenesis shows that the systemic influences cannot be considered as general promoters of growth in the form of mitotic stimuli. The fact that castration decreases the tumour incidence and oestrogen slightly increases it is evidence against the hypothesis of Jackson and Robson (1957) that oestrogenic hormones compete with carcinogens for the specific growth receptors. It seems rather that specific levels of reactivity to DMBA in the tissues of the target organs and of their stimulation by as yet undefined endocrine agents have to be postulated to explain the differential behaviour of vaginal stroma and vaginal epithelium in tumour formation. The vulva shows such a high responsiveness to DMBA that an influence of systemic factors has been obscured if it exists at all.

SUMMARY

Ovariectomy reduced the incidence of vaginal tumours after intravaginal application of DMBA, and administration of oestrogen or of progesterone raised the incidence of tumours only slightly.

Repeated whole body exposures to X-rays also lowered the rate of tumour incidence after painting and so to a lesser extent did repeated pelvic irradiation of virgin rats and the application of DMBA to an additional dorsal skin region.

In surgical castrates adrenalectomy or repeated pelvic irradiation restored the level of tumour incidence to that of intact and pregnant rats.

Three levels of vaginal tumour incidence are found and the distribution of tumour types and the length of the average induction time varied with the level: at the lowest level there are only sarcomas, at the intermediate level fibromas and presarcomatous lesions are found in addition to the sarcomas while at the highest level the incidence of sarcomas is increased and epithelial tumours appear.

Tumour induction in the vulva is not affected by castration, radiation or hormone treatment but varies at certain dose levels with the dose of the carcinogen.

The authors have pleasure in acknowledging their gratitude to Dr. H. B. Fell, F.R.S. for her constructive criticism of the manuscript, to Mr. G. C. Lenney for technical assistance, to Mr. H. G. Hignall for irradiating and Mr. G. W. Stebbings for looking after the animals.

This work was carried out under a grant from the British Empire Cancer Campaign.

REFERENCES

BIELSCHOWSKY, F. AND HORNING, E. S.—(1958) *Brit. med. Bull.*, **14**, 106.

GARDNER, W. U.—(1953) "Hormonal aspects of experimental tumorigenesis". *In* 'Advances in Cancer Research', Vol. 1. New York (Academic Press), p. 173.

GEYER, R. P., BRYANT, J. E. BLEISCH, V. R., PEIRCE, E. M. AND STARE, F. J.—(1953) *Cancer Res.*, **13**, 503.

GLUCKSMANN, A. AND CHERRY, C. P.—(1958) *Brit. J. Cancer*, **12**, 32.

*Idem*, LAMERTON, L. F. AND MAYNEORD, W. V.—(1957) "Carcinogenic effects of radiation". *In* Raven, R. W., 'Cancer', Vol. 1, 497. London (Butterworth & Co.).

GRIFFIN, A. C., RINFRET, A. P. AND CORSIGILIA, V. F. (1953) *Cancer Res.*, **13**, 77.

HALL, B. V., BALDER, R. N. JR. AND HAMILTON, K.—(1953) *Proc. Amer. Ass. Cancer Res.*, **1**, 23.

HOWELL, J. S., MARCHANT, J. AND ORR, J. W.—(1954) *Brit. J. Cancer*, **8**, 635.

HUGGINS, C., BRIZIARELLI, G. AND SUTTON, H.—(1959) *J. exp. Med.*, **109**, 25.

*Idem*, TORRALBA, Y. and MAINZER, K.—(1956) *Ibid.*, **104**, 525.

JACKSON, D. AND ROBSON, J. M.—(1957) *J. Endocrin.*, **14**, 348.

KIRSCHBAUM, A.—(1957) *Cancer Res.*, **17**, 432.

LISCO, H., DUCOFF, H. S. and BASERGA, R.—(1958) *Johns Hopk. Hosp. Bull.*, **103**, 101.

MARCHANT, J.—(1959) *Brit. J. Cancer*, **13**, 106.

MÜHLBOCK, O.—(1956) "The hormonal genesis of mammary cancer". *In* 'Advances in Cancer Research', Vol. 4. New York (Academic Press), p. 371.

SHAY, H., HARRIS, C. AND GRUENSTEIN, M.—(1952) *J. nat. Cancer Inst.*, **13**, 307.

SHIMKIN, M. B.—(1955) "Pulmonary tumours in experimental animals". *In* "Advances in Cancer Research", Vol. 3. New York (Academic Press), p. 223.

SLAWIKOWSKY, G. J. M.—(1960) *Cancer Res.*, **20**, 316.